# Learning curve for single-incision laparoscopic resection of right-sided colon cancer by complete mesocolic excision: Erratum

**DOI:** 10.1097/MD.0000000000005662

**Published:** 2016-12-09

In the article “Learning curve for single-incision laparoscopic resection of right-sided colon cancer by complete mesocolic excision”,^[1]^ which appeared in Volume 95, Issue 26 of *Medicine*, Dr. Yoon Dae Han's name was originally misspelled as Yun Dae Han.
